# Supplementary material for: Building blocks and blueprints for bacterial autolysins
Source: PLoS Comput Biol. 2021 Apr 1;17(4):e1008889. doi: 10.1371/journal.pcbi.1008889 (PMC8051824; doi:10.1371/journal.pcbi.1008889)
Supplement: S1 Fig — In each heatmap, each row and column represents a single non-redundant domain sequence in the LEDGOs database, and the cell for a pair of sequences is colored to indicate sequence identity (darker blue, higher). Cells are grouped by organism and clustered within an organism based on sequence identity patterns, so that similar sequences within an organism appear together as “blocks” on the diagonal, and blocks of similar sequences across organisms as off-diagonal blocks. Architectures of the associated sequences are indicated by row/column colors. (PDF) [file pcbi.1008889.s001.pdf]

The following legends hold for all figures; each figure has its own architecture legend (inner side colors).

organism (outer side colors) *% id (cell colors)*

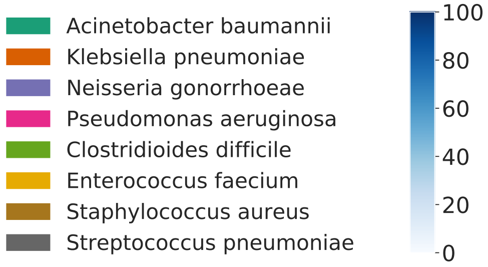

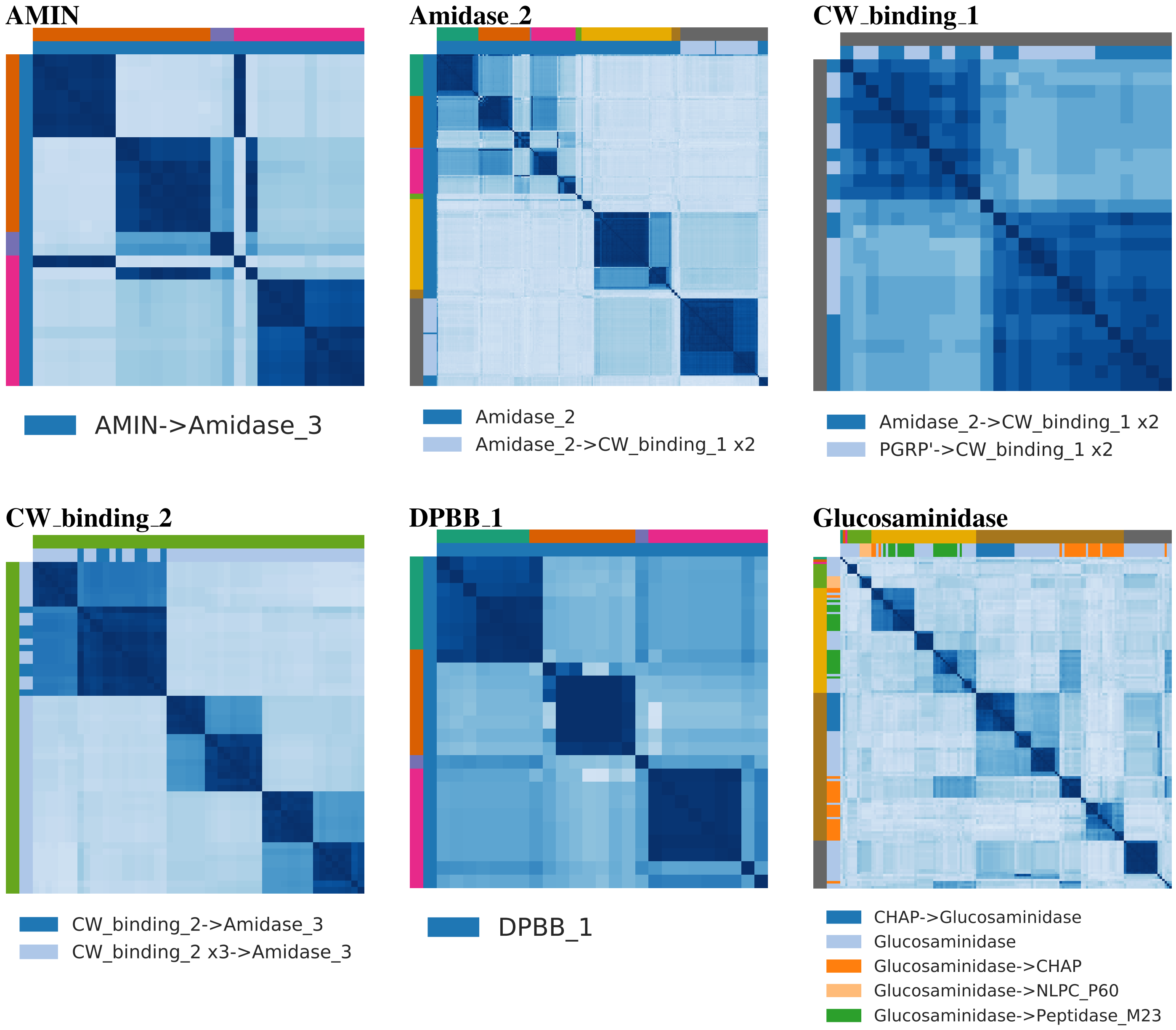
AMIN
AMIN->Amidase_3
Amidase_2
Amidase_2
Amidase_2->CW_binding_1 x2
CW_binding_1
Amidase_2->CW_binding_1 x2
PGRP'->CW_binding_1 x2
CW_binding_2
CW_binding_2->Amidase_3
CW_binding_2 x3->Amidase_3
DPBB_1
DPBB_1
Glucosaminidase
CHAP->Glucosaminidase
Glucosaminidase
Glucosaminidase->CHAP
Glucosaminidase->NLPC_P60
Glucosaminidase->Peptidase_M23

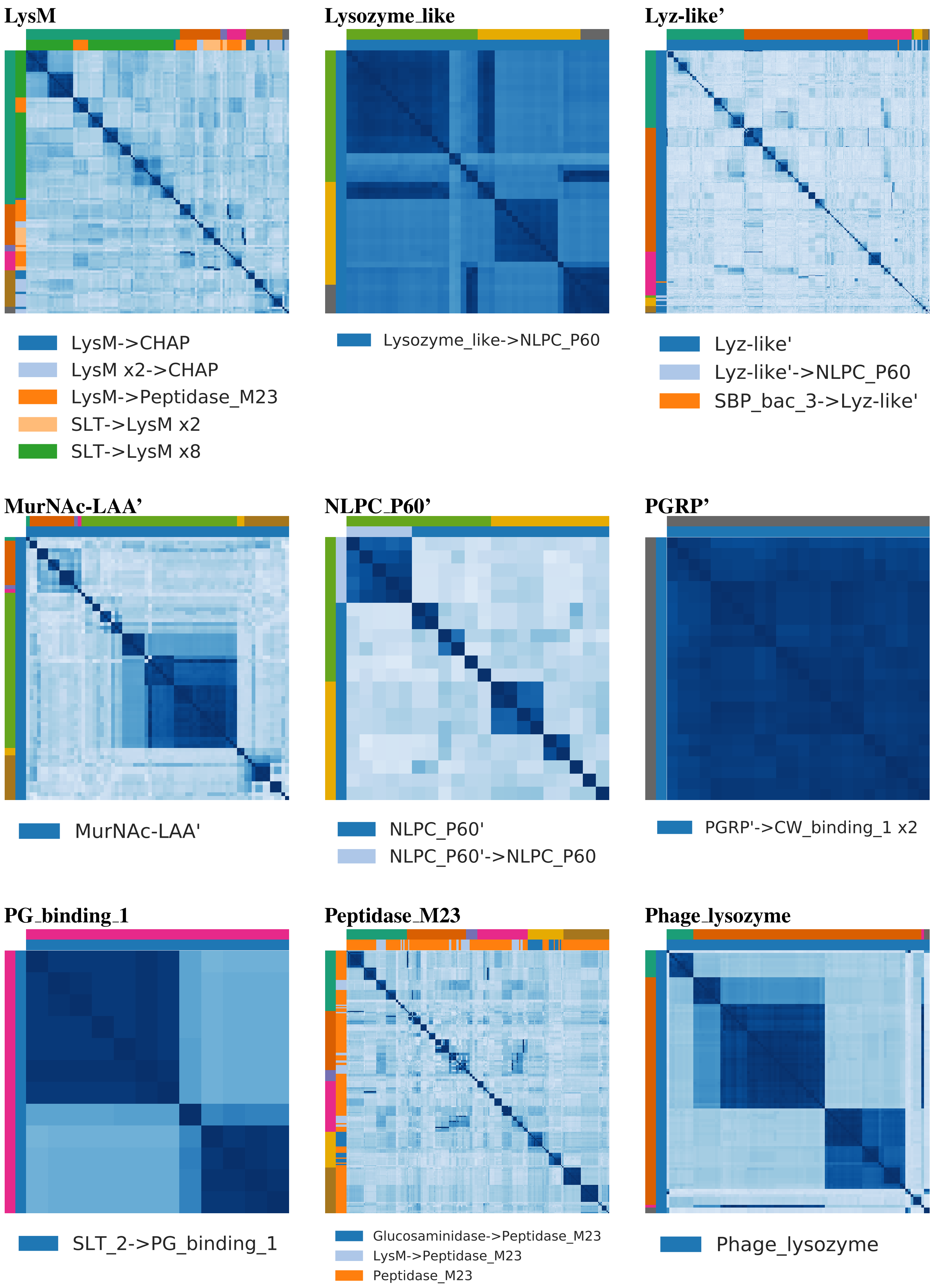

LysM
LysM->CHAP
LysM x2->CHAP
LysM->Peptidase_M23
SLT->LysM x2
SLT->LysM x8
Lysozyme_like
Lysozyme_like->NLPC_P60
Lyz-like’
Lyz-like'
Lyz-like'->NLPC_P60
SBP_bac_3->Lyz-like'
MurNAc-LAA’
MurNAc-LAA'
NLPC_P60’
NLPC_P60'
NLPC_P60'->NLPC_P60
PGRP’
PGRP'->CW_binding_1 x2
PG_binding_1
SLT_2->PG_binding_1
Peptidase_M23
Glucosaminidase->Peptidase_M23
LysM->Peptidase_M23
Peptidase_M23
Phage_lysozyme
Phage_lysozyme

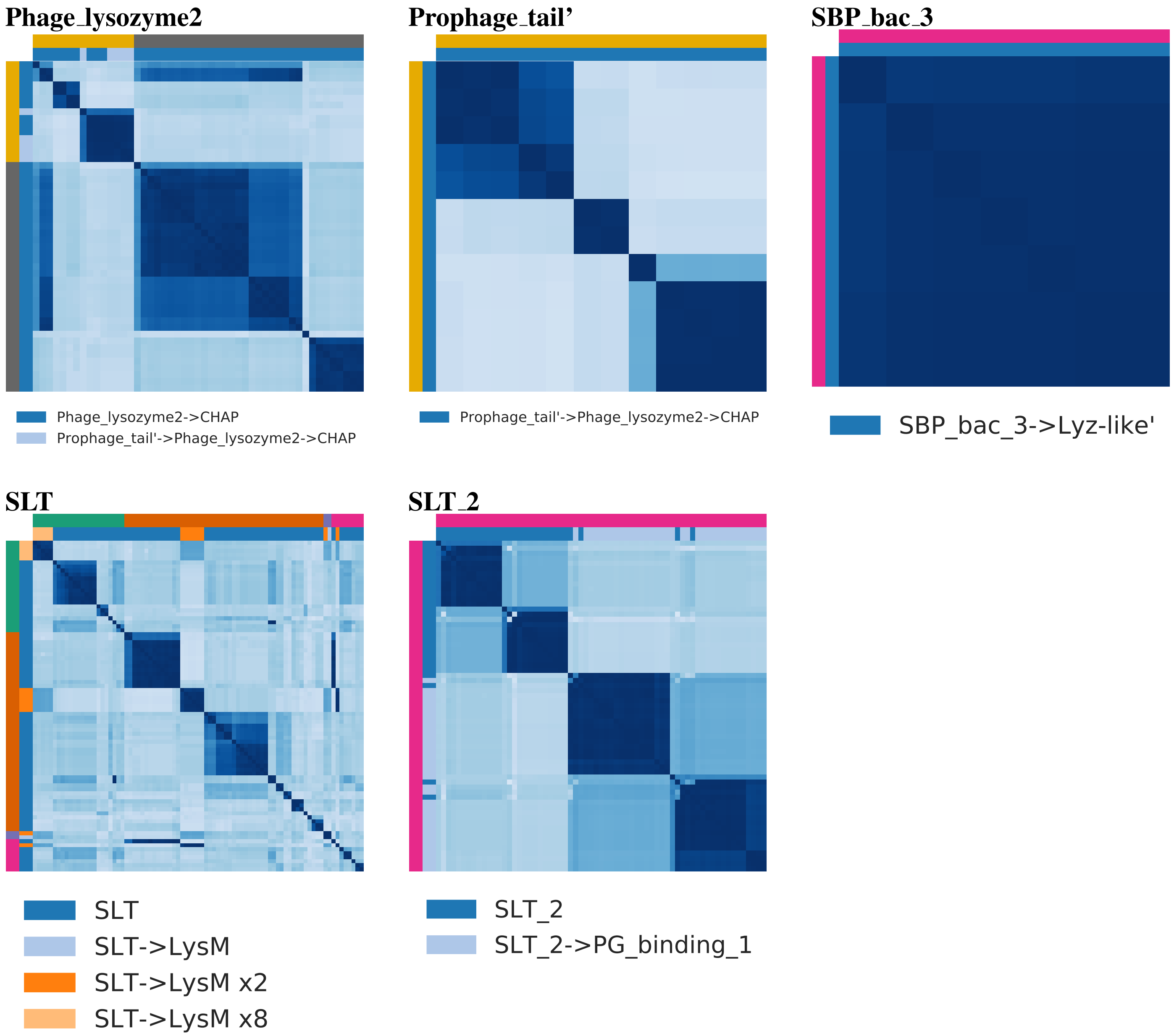
Phage_lysozyme2
Phage_lysozyme2->CHAP
Prophage_tail'->Phage_lysozyme2->CHAP
Prophage_tail'
Prophage_tail'->Phage_lysozyme2->CHAP
SBP_bac_3
SBP_bac_3->Lyz-like'
SLT
SLT
SLT->LysM
SLT->LysM x2
SLT->LysM x8
SLT_2
SLT_2
SLT_2->PG_binding_1
