# Supplementary material for: Building blocks and blueprints for bacterial autolysins
Source: PLoS Comput Biol. 2021 Apr 1;17(4):e1008889. doi: 10.1371/journal.pcbi.1008889 (PMC8051824; doi:10.1371/journal.pcbi.1008889)

(A)

```
-----------------------------------------------------------------------------
No. of Repeats|Total Score|Length  |Diagonal| BW-From|   BW-To|   Level
             2|     107.74|      34|     130|     202|     242|       5
-----------------------------------------------------------------------------
  202-  242 (51.34/46.08)      IWRSMLEHvnWNMIDNGvppkdKYEALE.KALFNIFKNREKL
  335-  369 (56.39/32.55)      IWESNTQC..YQMLNLG.....KYQGVSvSSLNKILKGKGTL
-----------------------------------------------------------------------------
```

(B)

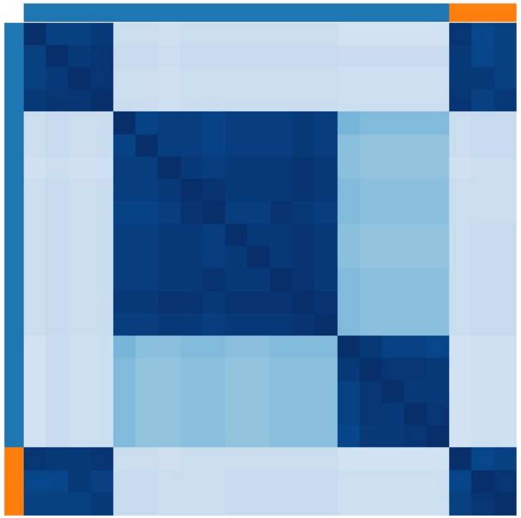

(C)

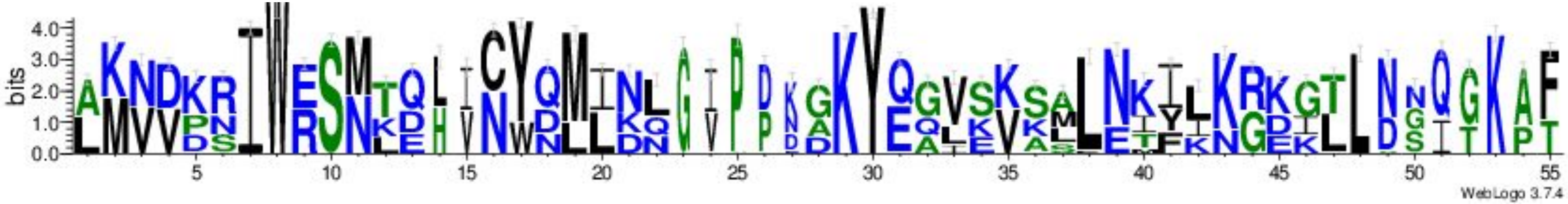

Supplement: S2 Fig — (A) Output from RADAR highlighting two sequence regions with similar sequences. (B) Sequence identity between RUF-8 repeat regions 1 (blue side color) and 2 (orange side color). (C) Sequence logo for the RUF-8 repeat sequences. (PDF) [file pcbi.1008889.s002.pdf]
