# Supplementary material for: Building blocks and blueprints for bacterial autolysins
Source: PLoS Comput Biol. 2021 Apr 1;17(4):e1008889. doi: 10.1371/journal.pcbi.1008889 (PMC8051824; doi:10.1371/journal.pcbi.1008889)
Supplement: S1 Table — (PDF) [file pcbi.1008889.s003.pdf]

S1 Table: Domain types used in the presented LEDGOs database.

Name	Accession	Type	Function
**CWB**: cell wall binding domains			
AMIN	pfam11741	Domain	CWB
CW_binding_1	pfam01473	Domain	CWB
CW_binding_2	pfam04122	Domain	CWB
LysM	pfam01476	Domain	CWB
PG_binding_1	pfam01471	Domain	CWB
SH3_3	pfam08239	Domain	CWB
SH3_5	pfam08460	Domain	CWB
SH3_8	pfam13457	Domain	CWB
SPOR	pfam05036	Domain	CWB
LysM	cl21525	Superfamily	CWB
**MurNAc-LAA**: aka N-acetylmuramyl-L-ala amidases, target the bond between MurNAc residues and L-alanine residues of the stem peptide			
Amidase_2	pfam01510	Domain	MurNAc-LAA
Amidase_3	pfam01520	Domain	MurNAc-LAA
MurNAc-LAA	cl02713	Superfamily	MurNAc-LAA
PGRP	cl02712	Superfamily	MurNAc-LAA
**N-acetylglucosaminidase**: aka endo-N-acetyl-beta-D-glucosaminidases, cleave the bond between GlcNAc and neighboring monosaccharides			
Glucosaminidase	pfam01832	Domain	N-acetylglucosaminidase
**N-acetylmuramidase**: aka N-acetyl-beta-D-muramidases, cleave the glycan backbone, targeting the bonds between MurNAc and GlcNAc			
DPBB_1	pfam03330	Domain	N-acetylmuramidase
Glyco_hydro_25	pfam01183	Domain	N-acetylmuramidase
Lysozyme_like	pfam13702	Domain	N-acetylmuramidase
Phage_lysozyme	pfam00959	Domain	N-acetylmuramidase
Phage_lysozyme2	pfam18013	Domain	N-acetylmuramidase
SLT	pfam01464	Domain	N-acetylmuramidase
SLT_2	pfam13406	Domain	N-acetylmuramidase
Lyz-like	cl00222	Superfamily	N-acetylmuramidase
**Peptidase**: target amide bonds between amino acids			
CHAP	pfam05257	Domain	Peptidase

Peptidase_M23	pfam01551	Domain	Peptidase
**Unknown catalytic**: CAT activity of uncharacterized specificity			
NLPC_P60	pfam00877	Domain	Unknown catalytic
NLPC_P60	cl21534	Superfamily	Unknown catalytic
**Other**: none of the above			
B	pfam02216	Domain	Other
ChW	pfam07538	Domain	Other
DUF2286	cl02234	Superfamily	Other
DUF4200	pfam13863	Domain	Other
GBS_Bsp-like	pfam08481	Domain	Other
Gram_pos_anchor	pfam00746	Domain	Other
Lactamase_B	pfam00753	Domain	Other
PhageMin_Tail	pfam10145	Domain	Other
RICH	pfam05062	Domain	Other
SBP_bac_3	pfam00497	Domain	Other
SpaA	pfam17802	Domain	Other
TonB_N	pfam16031	Domain	Other
YSIRK_signal	pfam04650	Domain	Other
Prophage_tail	cl23994	Superfamily	Other
Trep_Strep	cl09823	Superfamily	Other
metallo-hydrolase-like_MBL-fold	cl23716	Superfamily	Other
