# Supplementary material for: Building blocks and blueprints for bacterial autolysins
Source: PLoS Comput Biol. 2021 Apr 1;17(4):e1008889. doi: 10.1371/journal.pcbi.1008889 (PMC8051824; doi:10.1371/journal.pcbi.1008889)
Supplement: S2 Table — (PDF) [file pcbi.1008889.s004.pdf]

S2 Table: GO terms used in the presented LEDGOs database.

GO Term	GO ID
peptidoglycan binding	GO:0042834
peptidoglycan catabolic process	GO:0009253
N-acetylmuramoyl-L-alanine amidase activity	GO:0008745
peptidoglycan N-acetylglucosaminidase activity	GO:0061784
peptidoglycan muralytic activity	GO:0061783
lysozyme activity	GO:0003796
lytic transglycosylase activity	GO:0008933
lytic endotransglycosylase activity	GO:0008932
peptidoglycan endopeptidase activity	GO:0061785
peptidoglycan stem peptide endopeptidase activity	GO:0061786
peptidoglycan cross-bridge peptide endopeptidase activity	GO:0061787
