# Supplementary material for: Building blocks and blueprints for bacterial autolysins
Source: PLoS Comput Biol. 2021 Apr 1;17(4):e1008889. doi: 10.1371/journal.pcbi.1008889 (PMC8051824; doi:10.1371/journal.pcbi.1008889)
Supplement: S3 Table — (PDF) [file pcbi.1008889.s005.pdf]

S3 Table: Simplified LEDGOs database schema.

Organism
- Name
- Taxonomy ID
- Gram +/-

Protein
- Accession
- Fasta header/description
- Gene
- Sequence
- Organism

Superfamily
- Accession
- Short name
- Description
- CAT/CWB category

Domain
- Accession
- Short name
- Description
- Representative sequence
- CAT/CWB category
- Superfamily

RUF
- RUF ID
    - Unique generated identifier (e.g. RUF1, RUF2, etc…)
- Region ID
    - Unique identifier created from accession and start/stop positions in RUF representative.
- Representative sequence

Superfamily/Domain/RUF - Protein alignment
- Protein
- Superfamily/Domain/RUF
- Protein start/stop residues
- Superfamily/Domain/RUF start/stop residues
