# Supplementary material for: Building blocks and blueprints for bacterial autolysins
Source: PLoS Comput Biol. 2021 Apr 1;17(4):e1008889. doi: 10.1371/journal.pcbi.1008889 (PMC8051824; doi:10.1371/journal.pcbi.1008889)
Supplement: S4 Table — (PDF) [file pcbi.1008889.s006.pdf]

S4 Table: BLAST search of nr for selected RUFs

RUF ID	Representative Accession	Genera counts
RUF1	tr|A0A656DKU2|129-505	Streptococcus (1796)
RUF2	tr|A0A2A7T059|172-338	Enterococcus (982)
RUF3	tr|A0A5C8XB51|1-95	Staphylococcus (1820)
RUF4	tr|A0A140GY64|1-101	Staphylococcus (2068)
RUF5	tr|A0A1A7T323|78-219	Streptococcus (258), Lactobacillus (185), Enterococcus (142)
RUF6	tr|A0A450MA70|145-252	Clostridioides (133)
RUF7	tr|A0A133PXB4|1-263	Staphylococcus (933)
RUF8	tr|A0A6B5SNX0|121-492	Staphylococcus (1502)
RUF9	tr|A0A657S395|1-349	Salmonella (1019), Escherichia (656), Klebsiella (650), Enterobacter (234), Cronobacter (92), Citrobacter (76), Enterobacteriaceae (51)
RUF10	tr|A0A6B5T2J0|387-947	Staphylococcus (936)
RUF11	tr|A0A0E1PSR8|1-153	Acinetobacter (762)
RUF12	tr|A0A009FRV1|242-612	Acinetobacter (727)
RUF13	tr|L7PFM4|1-175	Clostridioides (832)
RUF14	tr|A0A1W6AZ89|1-431	Streptococcus (1823)
RUF15	tr|A0A6B5AU55|369-488	Staphylococcus (1166)
RUF16	tr|B0VNL7|104-384	Acinetobacter (541), Psychrobacter (62)
RUF17	tr|A0A656DUR5|97-419	Streptococcus (957)
RUF18	tr|A0A1W6AYZ0|1-549	Streptococcus (1039)
RUF19	tr|A0A655HBU9|480-627	Streptococcus (59)
RUF20	tr|A0A3F3NM44|1-434	Enterococcus (296)
RUF21	tr|A0A6B0CKL8|266-406	Staphylococcus (104)
