# Supplementary material for: Building blocks and blueprints for bacterial autolysins
Source: PLoS Comput Biol. 2021 Apr 1;17(4):e1008889. doi: 10.1371/journal.pcbi.1008889 (PMC8051824; doi:10.1371/journal.pcbi.1008889)
Supplement: Attachment — Submitted filename: response.docx [file pcbi.1008889.s007.docx]

We would like to thank the reviewers for their enthusiasm for our manuscript and their careful reading and helpful comments. We have addressed the comments in this revision, as shown in the change-tracked version and summarized here in blue below the reviewer comments.

### Reviewer #1

The manuscript by Mitchell et al. reports a new analysis tool for bacterial autolysins. The LEDGOs pipeline allows for a comparison of autolysins and their predicted domain architectures across bacterial species. This will be a highly useful tool for the field. The paper is well-written and informative and I only have minor comments.

We very much appreciate the reviewer’s support for our contribution, along with the following very helpful suggestions.

For simplicity, we aggregated these minor edits into a block:

1. Line 80 – spell out CHAP upon first use

3. Line 96 – for completeness’s sake, also cite the Gram-negative literature, e.g. PMID 23062283, 31286580, 26032134,

6. Line 198 spell out PGRP upon first use

7. Lines 207 and 209 – stay a bit more formal, i.e. spell out “Gram-negatives” and Gram-positives”

8. Line 213 spell out ChW domains upon first use.

We have made all of these corrections.

2. Line 92 and elsewhere – the authors often semantically conflate using autolysins as antibiotics with using them as novel antibiotic targets. In line 92, it should be antibiotic targets.

4. Line 100 should be “biotherapeutic target”

In the cases pointed out by the reviewer, we actually did mean “antibiotics” and “biotherapeutics”, rather than “antibiotic targets”, since it would be detrimental to the bacterium to evolve resistance to a drug that is also part of its own physiology. However we see how the wording was confusing, and have elaborated the text accordingly.

5. Line 119 etc: Please outline how these pathogens were prioritized for analysis vs. the more standard model organisms E. coli and B. subtilis (the ones they use are actually better, but for readability it should still be spelled out clearly why they abandoned the model organisms)

We have now made this selection clearer by stating that these pathogens are considered important in terms of their antibiotic resistance threat, making them good targets for novel therapies (both developing new antibiotics against their autolysins, as well as using their autolysins as novel antibiotics).

9. Line 281 seems like a contradiction – some domains occur in a variety of bacteria but appear to be specific to each? (specific suggests they only occur in certain bacteria, not a variety). Please make clearer.

We apologize for the confusing wording -- we meant that while they do occur in many bacteria, the sequences are so different across the bacteria that their specificities may also differ. We have reworded the text accordingly.

Also, as far as I know, NlpC_P60 enzymes occur in both Gram-positives and Gram-negatives (E. coli’s MepS is a P60 family enzyme – but maybe the annotation has simply changed now?).

Throughout the manuscript, we intend for the statements of general observations to be limited to only the 8 representative bacteria analyzed here. We added the word “these” to hopefully reduce the confusion / narrow the implication here.

10. Fig. 7 would be even more useful to the field if they gave representative gene names/uniprot accession for these RUFs

Good thought; we have added Supplementary Table 4 with that information (along with the information requested by reviewer 2 below).

### Reviewer #2

Mitchell et al. introduce LEDGOs, a pipeline to find and characterize autolysins with the aim to find new chemotherapeutic targets and to develop antibacterial biotherapies. The authors apply LEDGOs to eight different pathogens and following they describe and compare the results between the different organisms in regard to their domain composition, domain architecture as well as sequence diversity. Furthermore, they analyse interdomain regions of unknown function and group the potential new domains as rather catalytic or cell wall binding domains.

Thanks for this nice summary of our work and the following very helpful comments.

Introduction: The authors may want to introduce LEDGOs in more depth already in the introduction by differentiating between the LEDGOs pipeline and LEDGOs database and by explaining how beneficial this pipeline could be for the users.

This is a good thought, and goes hand-in-hand with the following suggestion for Fig. 1. We have updated the figure and text accordingly.

Fig. 1: It is useful to have an overview figure (Fig. 1), which explains how LEDGOs works but I may recommend re-structuring it. It is not clear which texts and images belong to the long arrow and particularly which part of the analysis is done by LEDGOs. Based on the current figure I would assume that I need all information (seed proteins, homologs, domains, architecture, RUFs) before running LEDGOs and not that this information will be stored in the LEDGOs database.

We see the source of confusion, and agree it’s beneficial to distinguish the user’s input (the seed sequences if desired, now indicated as the sole input), the LEDGOs data collection pipeline (now a straight arrow starting from below the input and going into the database), the resulting LEDGOs database, and the LEDGOs analyses (the arrows coming out of the database). We hope that the revised figure and text makes the pipeline and processes clearer.

Fig. 6: Is the legend description in figure 6 correct that the row colors indicate the repeat numbers and the column colors denote the architecture? I assumed that the inner left side is called ‘inner column’ and represents the repeat number and the inner top row is called ‘inner row’ and represents the architecture.

We apologize for the confusion; the heatmap package that we use calls the colors above the columns the “column side” colors, and likewise the “row side” colors are those next to the rows. We have reworded the caption to avoid the jargon.

Page 16, line 351-354: The authors may consider adding the DUF 2286 domain plus PFAM ID to the supplementary table S1. It might also be interesting to note that in PFAM, DUF 2286 is so far found only in archaea.

Thank you for pointing out the missed entry in the table; we have added it. We have also noted that the domain entry in Pfam is derived from archaeal sequences.

Fig. 7: Even though the analysis was conducted for different bacterial pathogens separately, it would be interesting to know in how many different bacterial genera the identified RUFs can be found and if the RUFs are found in other protein types besides autolysins.

This is a great point. We have updated the text and added Supplementary Table 4 to include the results of searching NCBI’s protein database for similar sequences.

Table 1: Remove repeats found with RADAR from s. aureus RUF counts.

Originally, the sub-sequences from figure S2 were included in the RUF count for *S. aureus*. For sake of consistency, we have now removed them.
